# Sorafenib in Combination with Betulinic Acid Synergistically Induces Cell Cycle Arrest and Inhibits Clonogenic Activity in Pancreatic Ductal Adenocarcinoma Cells

**DOI:** 10.3390/ijms19103234

**Published:** 2018-10-19

**Authors:** Justyna Kutkowska, Leon Strzadala, Andrzej Rapak

**Affiliations:** Department of Experimental Oncology, Ludwik Hirszfeld Institute of Immunology and Experimental Therapy Polish Academy of Science, 53-114 Wroclaw, Poland; justyna.kutkowska@iitd.pan.wroc.pl (J.K.); strzadal@iitd.pan.wroc.pl (L.S.)

**Keywords:** pancreatic ductal adenocarcinoma, combination therapy, sorafenib, betulinic acid, clonogenic activity

## Abstract

Pancreatic ductal adenocarcinoma (PDAC) is one of the most deadly cancers in the world due to late diagnosis and poor response to available treatments. It is important to identify treatment strategies that will increase the efficacy and reduce the toxicity of the currently used therapeutics. In this study, the PDAC cell lines AsPC-1, BxPC-3, and Capan-1 were treated with sorafenib and betulinic acid alone and in combination. We examined the effect of combined treatments on viability (MTS test), proliferation and apoptosis (annexin V staining), cell cycle arrest (PI staining), alterations in signaling pathways (Western blotting), and colony-forming ability. The combination of sorafenib with betulinic acid inhibited the viability and proliferation of PDAC cells without the induction of apoptosis. The antiproliferative effect, caused by G2 cell cycle arrest, was strongly associated with increased expression of p21 and decreased expression of c-Myc and cyclin D1, and was induced only by combined treatment. Additionally, decreased proliferation could also be associated with the inhibition of the P13K/Akt and MAPK signaling pathways. Importantly, combination treatment reduced the colony-forming ability of PDAC cells, as compared to both compounds alone. Collectively, we showed that combined treatment with low concentrations of sorafenib and betulinic acid had the capacity to inhibit proliferation and abolish clonogenic activity in PDAC cell lines.

## 1. Introduction

Pancreatic ductal adenocarcinoma (PDAC) is one of the most deadly cancers of the digestive system worldwide [1]. Pancreatic cancer affects both men and women, and the overall five-year survival rate remains below 5% [2]. Chemotherapy is an important therapeutic method, but the sensitivity is low due to increasing drug resistance [3]. This situation is probably caused by the specific tumor microenvironment of pancreatic cancer.

Currently, gemcitabine is used as a standard therapy for advanced pancreatic cancer. However, gemcitabine alone is not very effective and is associated with drug resistance [4]. In view of that problem, developing new agents and innovative approaches are a continuing research effort to advance the treatment of this disease [5].

We have previously shown that combination treatment with sorafenib (SOR) and betulinic acid (BA) inhibits proliferation, induces cell death, and reduces colony-forming ability in non-small cell lung cancer (NSCLC) cell lines with different KRAS mutations [6]. In pancreatic cancer, activating KRAS mutations occur at a frequency of 90% [7]. Previous studies have shown that almost all therapies targeting KRAS mutations have failed [8]. Many efforts have been made in the field of PDAC therapies to develop drugs or combination of drugs targeting the components of the downstream effector pathways of KRAS signals, such as the MAPK and PI3K/Akt signaling pathways [9].

Sorafenib (SOR) is an oral, multitargeted kinase inhibitor directed against the mitogen-activated protein kinase (MAPK) pathway, vascular endothelial growth factor receptor-2 (VEGFR-2) and -3 (VEGFR-3), platelet-derived growth factor receptor-b (PDGFRb), Fms-related tyrosine kinase 3 (FLT3), and mast/stem cell growth factor receptor Kit (c-KIT) [10,11]. Sorafenib is approved for the treatment of renal cell carcinoma, hepatocellular carcinoma, and thyroid cancer [12,13]. The data suggest that sorafenib has a potential therapeutic benefit for PDAC treatment; however, the results of a combination therapy utilizing sorafenib with gemcitabine or sorafenib with erlotinib indicated that sorafenib was not able to enhance chemotherapeutic effect [14,15]. 

Several anticancer and anti-infectious drugs are derived from natural products [16]. Betulinic acid (BA) is a natural pentacyclic triterpene with a lupine structure isolated from the bark of the white birch (*Betula pubescens*) [17]. BA has been shown to induce apoptosis in a p53- and caspase-independent manner, mitochondrial membrane alteration, and DNA fragmentation [18]. Importantly, some reports showed that BA induced cell death by downregulating the expression of the P13K/Akt signaling pathway [19]. BA exhibits significant antitumor activities in various cancer cells, including pancreatic cancer [20].

In this study, we showed that combination treatment with SOR and BA also reduced the clonogenic potential of PDAC cells, suppressed proliferation via cell cycle arrest, and inhibited the PI3K/Akt and MAPK signaling pathways, but it did not induce apoptosis. Combination therapies that act on different molecular targets in the cancer should increase the probability of cancer elimination and decrease the development of resistant cancer cells.

## 2. Results

### 2.1. The Combination of Sorafenib and Betulinic Acid Inhibits the Proliferation of PDAC Cell Lines, but Does Not Induce Apoptosis

**Figure 1:**
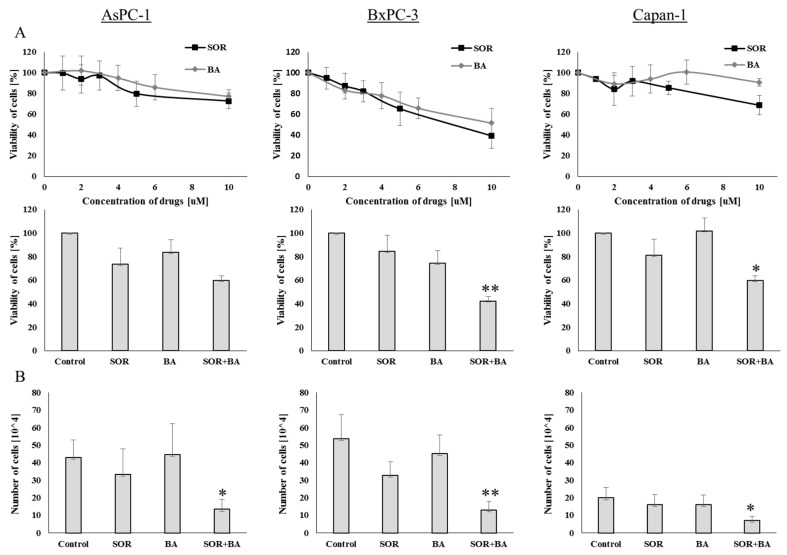
Effect of combination treatment with sorafenib (SOR) and betulinic acid (BA) on cell growth inhibition of PDAC cell lines. (**A**) Viability of AsPC-1, BxPC-3, and Capan-1 cells after exposure to sorafenib and betulinic acid at different drug concentrations (AsPC-1 and Capan-1: 5 µM, BxPC-3: 3 µM) and betulinic acid (6 µM) alone and in combination. (**B**) The number of AsPC-1, BxPC-3, and Capan-1 cells after treatments with sorafenib (AsPC-1 and Capan-1: 5 µM, BxPC-3: 3 µM) and betulinic acid (6 µM) alone and in combination (*n* = 4). Data are presented as means ± SD normalized to the untreated control. * *p* < 0.05, ** *p* < 0.01 compared with the sorafenib treatment group and betulinic acid treatment group.

Recently, we have shown that combination treatment with SOR and BA caused significant proliferation-inhibitory effects in NSCLC cell lines [6]. We first investigated the effects of SOR and BA on cell viability using the MTS assay to determine whether their combination can inhibit the proliferation of human PDAC cell lines with different mutational KRAS status (Table 1). Treatment of BxPC-3 cells with SOR and BA resulted in dose-dependent growth inhibition, but two other cell lines, AsPC-1 and Capan-1, were resistant to treatment with SOR and BA after 72 h. Next, PDAC cell lines were treated with sorafenib (AsPC-1 and Capan-1, 5 µM; BxPC-3, 3 µM) and betulinic acid (6 µM) alone or in combination for 72 h. SOR and BA alone decreased cell viability by an average of 73.4 ± 13.6% and 83.5 ± 10.8% in AsPC-1 cells, 84.8 ± 10.9% and 72.3 ± 13.2% in BxPC-3 cells, 81.1 ± 12.2% and 101.8 ± 8.7% in Capan-1 cells, respectively, but combination treatment reduced cell viability more effectively to 59.8 ± 3.7% (CI = 0.829) in AsPC-1 cells, 42.2 ± 10.1% (CI = 0.633) in BxPC-3 cells, and 59.7 ± 5.7% (CI = 0.409) in Capan-1 cells (Figure 1A).

We subsequently investigated whether the combination of SOR and BA could inhibit PDAC cell proliferation using trypan blue staining. As shown in Figure 1B, combination treatment significantly reduced the number of cells from 45.5 ± 12.7 × 10^4^ to 13.3 ± 5.5 × 10^4^ (CI = 0.229) in AsPC-1 cells, from 46.8 ± 8.2 × 10^4^ to 13.02 ± 4.9 × 10^4^ (CI = 0.198) in BxPC-3 cells, and from 18.2 ± 3.8 × 10^4^ to 6.9 ± 2.5 × 10^4^ (CI = 0.272) in Capan-1 cells, as compared to either compound alone (*p* < 0.05).

**Figure 2:**
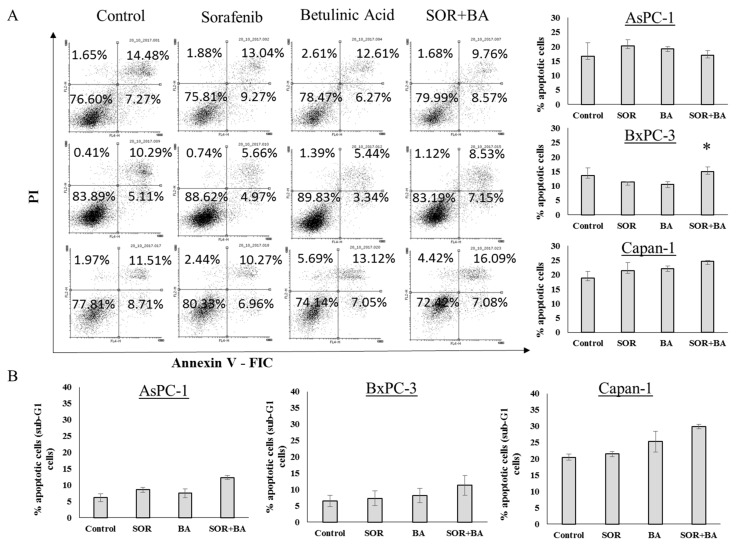
Cytotoxicity effect of combination treatment with SOR and BA on PDAC cells. (**A**) Representative FACS dot plots showing the effect of combination treatment with sorafenib (AsPC-1 and Capan-1: 5 µM, BxPC-3: 3 µM) and betulinic acid (6 µM) on phosphatidylserine exposure and plasma membrane integrity after 72 h of incubation with pancreatic cancer cells, as determined by annexin V-FIC/PI staining. (**B**) Apoptosis-associated DNA fragmentation of AsPC-1, BxPC-3, and Capan-1 cells after treatments with sorafenib (AsPC-1 and Capan-1: 5 µM, BxPC-3: 3 µM) and betulinic acid (6 µM) alone and in combination (*n* = 3). Data are presented as means ± SD. * *p* < 0.05 compared with the sorafenib treatment group and betulinic acid treatment group.

Additionally, we used the annexin V-FIC/PI double staining and apoptosis-associated DNA fragmentation by staining cells with propidium iodide (PI) to evaluate whether the SOR and BA combination induced apoptosis in PDAC cells. As shown in Figure 2, combination treatment did not increase apoptosis in PDAC cell lines.

### 2.2. The Combination of Sorafenib and Betulinic Acid Induces G2 Cell Cycle Arrest in AsPC-1 Cells

**Figure 3:**
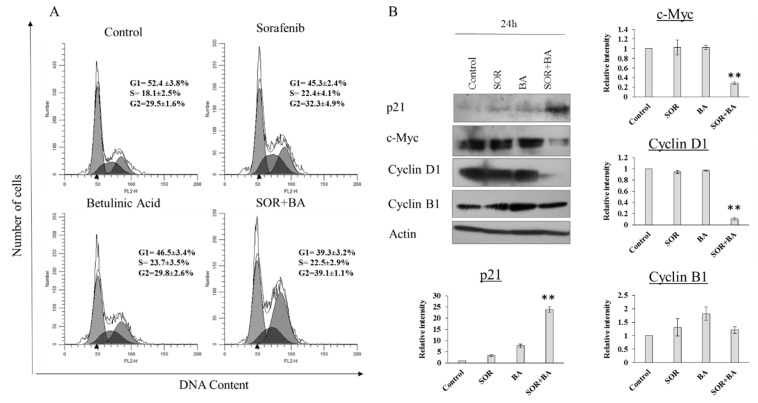
Effect of combination treatment with SOR and BA on cell cycle arrest in AsPC-1 cells. (**A**) Representative cell cycle analyzed by FACS of AsPC-1 cells after treatments with sorafenib (5 µM) and betulinic acid (6 µM) alone and in combination (*n* = 3). (**B**) Representative immunoblot of p21, c-Myc, cyclin D1, and cyclin B1 expression from AsPC-1 cells treated with sorafenib (5 µM) and betulinic acid (6 µM) alone and in combination (*n* = 3). Actin served as a loading control. Data are presented as means ± SD. * *p* < 0.05, ** *p* < 0.01 compared with the sorafenib treatment group and betulinic acid treatment group. All experiments were repeated at least three times.

The cell cycle distribution analysis was performed using flow cytometry to elucidate how the combination of SOR and BA inhibited cell proliferation. The results showed that the combination of SOR and BA significantly induced cell cycle arrest at G2 phase (Figure 3A). The percentage of G2 phase cells increased to 39% after treatment with the SOR and BA combination.

The effect was further confirmed by the detection of key proteins that help regulate the cell cycle. Figure 3B shows that the level of p21 increased after treatment with SOR and BA alone and in combination for 24 h, while the levels of c-Myc and cyclin D1 decreased after combination treatment. However, the expression of cyclin B1 remained unchanged. These results suggest that cell cycle arrest in the G2 phase is a probable mechanism by which SOR + BA prevent PDAC cell proliferation. The results were similar in the other two cell lines.

### 2.3. Combination Treatment with Sorafenib and Betulinic Acid Inhibits the Expression of the PI3K/Akt and MAPK Signaling Pathways in the AsPC-1 and BxPC-3 Cell Lines

**Figure 4:**
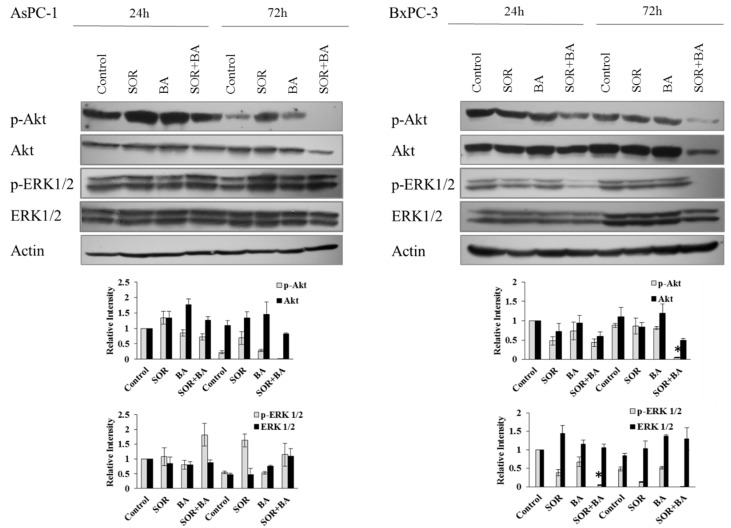
The effect of combination treatment with SOR and BA on protein expression of the PI3K/Akt and MAPK signaling pathways in human PDAC cell lines—AsPC-1 and BxPC-3. Representative immunoblot of phospho-Akt (Ser473), Akt, phospho-ERK1/2, and ERK1/2 expression from AsPC-1 and BxPC-3 cells treated with sorafenib (AsPC-1: 5 µM, BxPC-3: 3 µM) and betulinic acid (6 µM) alone and in combination (*n* = 3). Actin served as a loading control. Data are presented as means ± SD. * *p* < 0.05 compared with the sorafenib treatment group and betulinic acid treatment group.

We investigated the effects of SOR and BA alone and in combination on the PI3K/Akt and/or MAPK signaling pathways in AsPC-1 and BxPC-3 cells, because the activation of these pathways is important for cell cycle progression in human pancreatic cancer cells [23,24]. Western blotting results showed (Figure 4) that combination treatment inhibited ERK1/2 phosphorylation after 24 and 72 h in BxPC-3 cells. In addition, combination treatment inhibited the expression and phosphorylation of Akt after 72 h in AsPC-1 cells and after 24 and 72 h in BxPC-3 cells.

### 2.4. The Combination of Sorafenib and Betulinic Acid Reduces the Colony-Forming Ability of PDAC Cell Lines

**Figure 5:**
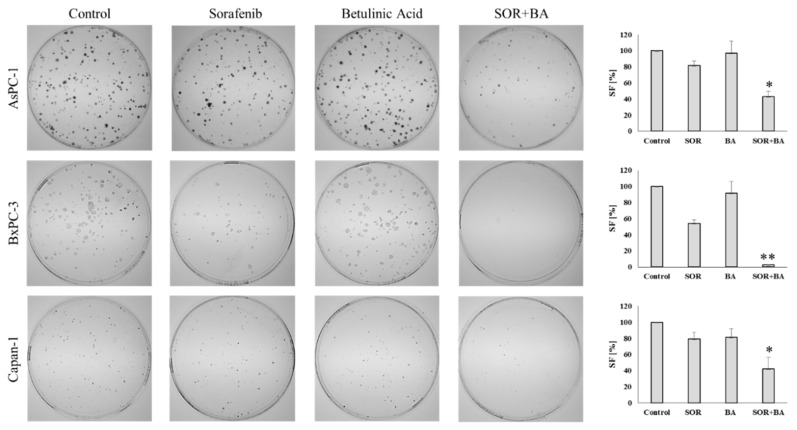
Effect of combination treatment with SOR and BA on the colony-forming ability of AsPC-1, BxPC-3, and Capan-1 cell lines. On the left: representative images of colonies formed by AsPC-1, BxPC-3, and Capan-1 cells after treatment with sorafenib (AsPC-1 and Capan-1: 5 µM, BxPC-3: 3 µM) and betulinic acid (6 µM) alone and in combination. On the right: the surviving fraction (SF) of AsPC-1, BxPC-3, and Capan-1 cells after treatment with sorafenib (AsPC-1 and Capan-1: 5 µM, BxPC-3: 3 µM) and betulinic acid (6 µM) alone and in combination (*n* = 3). * *p* < 0.05 ** *p* < 0.01 compared with the sorafenib treatment group and betulinic acid treatment group.

The long-term assay (clonogenic survival) was employed to determine the ability of combination treatment with SOR and BA to influence pancreatic cancer cell survival. PDAC cell lines were treated with sorafenib (AsPC-1 and Capan-1: 5 µM, BxPC-3: 3 µM) and betulinic acid (6 µM) alone or in combination for 14 days, after which the number of colonies formed was counted (Figure 5). The SOR and BA combination significantly reduced the number of colonies (surviving fraction) compared to the control and single sorafenib and betulinic acid treatments for all cell lines. In addition to reducing the number of colonies, colony size appeared to be smaller in the SOR+BA treatment, suggesting that the combination also prevented the clonogenic expansion of existing tumor cells.

## 3. Discussion

There are limited effective treatments for patients with pancreatic cancer. Gemcitabine combined with other adjuvants has been used to treat PDAC, but these approaches have had limited success and only extended life span by months with additional toxicity [25,26]. Several combination therapies with sorafenib, such as SOR with gemcitabine and erlotinib, have been evaluated in clinics; however, the outcomes were disappointing [14,15,27,28]. In addition, SOR has already been shown to exert no benefit for survival or other efficacy parameters in locally advanced or metastatic pancreatic adenocarcinoma [29]. Furthermore, Pandita et al. [20] showed that the combination of betulinic acid and gemcitabine inhibited cell proliferation, induced apoptosis, and downregulated the expression of PKM2 in PDAC cell lines. Moreover, betulinic acid was shown to have broad antitumor effects on PDAC cells in some other studies [30,31,32].

In this study, we determined that the natural product betulinic acid enhanced the effects of sorafenib in PDAC cell lines. Single treatment with SOR or BA shows dose-dependent growth inhibition only in the BxPC-3 cell line, whereas AsPC-1 and Capan-1 are highly resistant. Increased sensitivity of BxPC-3 cells can be caused by the presence of a mutation in BRAF, which is a possible target of sorafenib. We found that SOR in combination with BA significantly inhibited the proliferation, promoted cell cycle arrest, and reduced the colony-forming ability of PDAC cells in vitro. Interestingly, this combination did not induce apoptosis.

Our data showed that combination treatment with SOR and BA increased the expression of p21 and simultaneously decreased the expression of c-Myc and cyclin D1. Some authors also reported that cyclin D1 was involved in G2 arrest, especially under high levels of oxidative stress [33]. Hitomi et al. suggested that the elevated cyclin D1 level in the G2 phase was a critical checkpoint for the progression of the cell cycle [34]. Suppression of cyclin D1 levels during the G2 phase promoted the inhibition of proliferation [35]. The c-MYC proto-oncogene is activated in many PDAC cases and plays a central role in many cellular processes, such as proliferation, differentiation, and apoptosis [36]. Moreover, c-Myc overexpression was reported to be associated with gemcitabine resistance [37]. According to some reports, c-Myc inhibition was not always associated with cell death [38]; in vivo, c-Myc correlated inversely with p21 expression. The level of c-Myc determines if p21 is induced or suppressed and whether cells undergo apoptosis or growth arrest [39]. Increased p21 expression is associated with cell cycle arrest, proliferation inhibition, and cell senescence [40]. Cell cycle regulators, including p21, p27, and cyclins, should be tightly controlled; p21 coordinates with p27, thereby modulating the expression of cyclin D1 and E2 [40]. Deregulation of cyclin D1 can lead to genetic instability in in vitro and in vivo tumorigenesis [41]. Additionally, cyclin D1 overexpression is an independent prognostic factor for survival in patients with PDAC [42].

PI3K/Akt and MAPK/ERK are two of the early signaling pathways of cell cycle progression [28,43]. In this study, we demonstrated that combination treatment with SOR and BA inhibited pancreatic cancer cell cycle progression by inactivating PI3K/Akt and MAPK signaling. Recently published data have shown a synergic effect between sorafenib and HS-173 (a novel PI3K inhibitor). This treatment, by synergistically inhibiting the MAPK and PI3K/Akt pathways, induced G2/M arrest and increased apoptosis in pancreatic cancer cells [44]. However, while concurrent treatment with MEK and PI3K inhibitors has recently been investigated in mouse models of pancreatic cancer, only low antitumor activity was observed [45,46]. Moreover, a clinical trial of MEK and PI3K inhibitor combinations also suggested that normal tissue toxicity may limit this combination [40]. A clinical study of Akt and ERK inhibitor combinations needs to be conducted to determine whether targeting ERK rather than MEK will overcome these limitations.

Mechanisms of growth suppression by signaling inhibitors are usually characterized using short-term analyses, yet clinical application of such inhibitors involves persistent long-term treatment. In the present study, we performed a long-term clonogenic survival assay. Combination treatment with SOR and BA markedly decreased the colony-forming capability of PDAC cells. This treatment has been shown previously to be nontoxic for normal human peripheral blood lymphocytes [6]. It should be noted that tumor cell cloning efficiency is positively correlated with proliferation and self-renewal abilities, which may be associated with cell tumorigenesis [47]. Studying tumor clonogenic/stem-like cells contributes to the identification of molecular targets important in successful cancer therapy [48].

In summary, we have demonstrated for the first time that combination treatment with SOR and BA can more effectively attenuate cell proliferation, promote cell cycle arrest, and reduce colony-forming ability than a single treatment in human PDAC cell lines. Moreover, combined treatment with SOR and BA can inhibit phosphorylation of Akt and ERK1/2 more potently than their individual use, which may account for the synergistic antitumor effect of this combination treatment. This study may provide a novel indication for pancreatic cancer treatment.

## 4. Materials and Methods

### 4.1. Cell Culture and Reagents

Pancreatic ductal adenocarcinoma lines with different types of mutations (AsPC-1, BxPC-3, and Capan-1) were purchased from the American Type Culture Collection (Manassas, VA, USA) and cultured in the recommended growth media with 10% FBS (Gibco, Thermo Fisher Scientific, Waltham, MA, USA) and antibiotic/antimycotic solution (Sigma-Aldrich, St. Louis, MO, USA). All cell lines were cultured at 37 °C in a humidified atmosphere of 5% CO_2_. The cells were seeded at densities of 1 × 10^4^ cells/0.1 mL (0.32 cm^2^) (cell viability assay), 6 × 10^4^ cells/0.5 mL (1.9 cm^2^) (flow cytometry), 1 × 10^5^ cells/3 mL (9.5 cm^2^) (long-term colony formation assay, serial replating assay), and 1 × 10^6^ cells/4 mL (21 cm^2^) (Western blotting). The cells were treated with sorafenib (LC Laboratories), betulinic acid (Sigma-Aldrich Chemistry, St. Louis, MO, USA), and both at one day post-seeding. The cells were collected for the appropriate assay three days later.

### 4.2. Cell Viability Assay

Cell viability was assessed by the CellTiter 96 AQueous One Solution Cell Proliferation Assay (Promega, Madison, WI, USA), according to the manufacturer’s protocol. Each treatment within a single experiment was performed in triplicate. Absorbance at 490 nm was recorded using a Wallac 1420 VICTOR2 plate reader (PerkinElmer, Waltham, MA, USA). Data were normalized to the untreated control.

### 4.3. Cell Count

Floating and trypsinized cells were collected and suspended in fresh medium at room temperature. Twenty microliters of cell suspension was mixed with 20 μL of 0.4% Trypan blue solution (Bio-Rad, Carlsbad, CA, USA), and 20 μL of this mixture was used to count blue (dead) and white (alive) cells.

### 4.4. Analysis of Drug Interaction

The nature of the interactions between the drugs studied was analyzed with the help of combination-index (CI) methods, derived from the median-effect principle of Chou and Talalay [49]. CI values indicate the following: <0.1, very strong synergism; 0.1–0.3, strong synergism; 0.3–0.7, synergism; 0.7–0.85, moderate synergism; 0.85–0.9, slight synergism; 0.9–1.1, nearly additive; 1.1–1.2, slight antagonism; 1.2–1.45, moderate antagonism; 1.45–3.3, antagonism; 3.3–10, strong antagonism; >10, very strong antagonism. The CI value was calculated using CompuSyn software (ComboSyn, Inc., Paramus, NJ, USA). The CI was defined as follows: CI = (D)1/(Dx)1 + (D)2/(Dx)2 for mutually exclusive drugs. In the denominator, (Dx) is for D1 ‘‘alone’’ that inhibits a system x%, and (Dx)2 is for D2 ‘‘alone’’ that inhibits a system x%. In the numerators, (D)1 + (D)2 ‘‘in combination’’ also inhibit x%.

### 4.5. Annexin V Staining

Apoptosis was assessed by the Annexin V Apoptosis Detection Kit (Santa Cruz Biotechnology, Dallas, TX, USA), according to the manufacturer’s protocol. Briefly, the cells were stained with annexin V–FITC (8 μg/mL) and PI (5 μg/mL) for 15 min at RT in the dark. The cells were washed with cold PBS (with Ca^2+^ and Mg^2+^) containing 2.5% FBS between the steps. Data were acquired using a FACSCalibur flow cytometer (Becton Dickinson, Franklin Lakes, NJ, USA) and analyzed using Flowing Software 2.5.1 (Perttu Terho, Turku, Finland). Apoptosis was quantified as a percentage of both annexin V-positive and annexin V/PI-double-positive cells.

### 4.6. Cell Cycle and DNA Fragmentation Assay

The cells were fixed in 75% ethanol at 4 °C for 30 min, and then incubated with 50 ng/mL PI staining solution and 0.2 mg/mL RNase in the dark overnight at 4 °C. Data were acquired using a FACSCalibur flow cytometer (Becton Dickinson, Franklin Lakes, NJ, USA) and analyzed using the ModFit LT 5.0 software (Verity Software House, Inc., Topsham, ME, USA).

### 4.7. Western Blotting

Whole cell lysates were prepared using cold RIPA buffer (150 mM NaCl (POCH), 50 mM Tris–HCl pH 8.0 (BioShop, Burlington, ON, Canada), 1% NP-40 (Calbiochem, San Diego, CA, USA), 0.5% sodium deoxycholate (Sigma-Aldrich, St. Louis, MO, USA), and 1% SDS (BioShop, Burlington, ON, Canada)) supplemented with SigmaFAST Protease Inhibitor Cocktail (Sigma-Aldrich, St. Louis, MO, USA) and Halt Phosphatase Inhibitor Cocktail (Thermo Fisher Scientific, Waltham, MA, USA). Cell lysates were then sonicated for 10 s at 100% power using a Sonopuls HD 2070 ultrasonic homogenizer (Bandelin, Berlin, Germany) and centrifuged at 10,000× *g* for 10 min at 4 °C to pellet cellular debris. Protein concentration was determined by the Pierce BCA Protein Assay Kit (Thermo Fisher Scientific, Waltham, MA, USA), according to the manufacturer’s protocol. Absorbance at 570 nm was recorded using a Wallac 1420 VICTOR2 plate reader. Cell lysates with Laemmli sample buffer (50 mM Tris–HCl pH 6.8, 10% glycerol (BioShop, Burlington, ON, Canada), 5% 2-mercaptoethanol (Sigma-Aldrich, St. Louis, MO, USA ), 2% SDS, 0.05% bromophenol blue (BioShop, Burlington, ON, Canada)) were heated for 5 min at 95 °C, the proteins were separated by SDS-PAGE using 8–12% resolving gels (SDS-PAGE running buffer: 25 mM Tris, 192 mM glycine (BioShop Burlington, ON, Canada), 0.1% SDS) and transferred (semidry transfer) to a PVDF membrane (0.45 μm pore size; Merck Millipore) (transfer buffer: 25 mM Tris, 192 mM glycine, and either 10% or 20% methanol (POCH)). In between the steps, membranes were washed with TBST (20 mM Tris, 150 mM NaCl, 0.1% Tween 20 (BioShop, Burlington, ON, Canada)). Membranes were blocked either with 1% casein (0.1 M Tris–HCl pH 8.0, 214 mM NaCl, 1% casein from bovine milk (Sigma-Aldrich, St. Louis, MO, USA)) or 5% BSA/TBST (Sigma-Aldrich, St. Louis, MO, USA) for an hour at RT or overnight at 4 °C and then incubated with primary antibody overnight at 4 °C. After probing with HRP-conjugated secondary antibody for 1 h at RT, proteins of interest were detected using SuperSignal West Dura Extended Duration Substrate (Thermo Fisher Scientific, Waltham, MA, USA). The following antibodies were used in this study: anti-p21/HRP (1:1000, #sc-6246; Santa Cruz Biotechnology, Dallas, TX, USA), anti-c-Myc (1:1000, #sc-788; Santa Cruz Biotechnology, Dallas, TX, USA), anti-cyclin D1 (1:1000, #2978; Cell Signaling Technology, Danvers, MA, USA), anti-cyclin B1 (1:2000, #4135; Cell Signaling Technology, Danvers, MA, USA), anti-Akt (1:1000, #4691; Cell Signaling Technology, Danvers, MA, USA), anti-phospho-Akt (1:1000, #4060; Cell Signaling Technology, Danvers, MA, USA), anti-ERK1/2 (1:1000, #9102; Cell Signaling Technology, Danvers, MA, USA), anti-phospho-ERK1/2 (1:1000, #9101; Cell Signaling Technology, Danvers, MA, USA), anti-actin/HRP (1:2000, #sc-1615; Santa Cruz Biotechnology, Dallas, TX, USA), anti-mouse/HRP (1:2000, #P0447; Dako, Glostrup, Denmark), and anti-rabbit/HRP (1:2000–3000, #P0048; Dako, Glostrup, Denmark).

### 4.8. Long-Term Colony-Formation Assay

Viable cells were counted using the trypan blue method and seeded in duplicate at a density of 5 × 10^2^ cells/6 mL (21 cm^2^). The dishes had been precoated with poly-L-lysine/PBS (0.001%; Sigma-Aldrich, St. Louis, MO, USA) and washed twice with PBS (with Ca^2+^ and Mg^2+^). After 2 weeks, the colonies were fixed and stained with 1% crystal violet/ethanol (Sigma-Aldrich, St. Louis, MO, USA), documented with an Olympus Stylus SH-50 camera (Olympus, Tokyo, Japan), and counted manually using the ImageJ 1.47 software (National Institutes of Health, Bethesda, MD, USA). The term plating efficiency (PE) indicates the percentage of seeded cells that grow to form colonies. The surviving fraction (SF) is calculated as the ratio between the PEs of treated and control cells multiplied by 100.

### 4.9. Statistical Analysis

Data are presented as means ± SD of the results from at least three independent experiments. Comparisons between the two groups: sorafenib treatment group vs. combinatorial treatment group, and betulinic acid treatment group vs. combinatorial treatment group, were analyzed by a two-tailed Student’s *t*-test. The significance was assumed at * *p* < 0.05, ** *p* < 0.01.

## Figures and Tables

**Table 1:** Mutational status of pancreatic ductal adenocarcinoma (PDAC) critical genes [21,22].

PDAC Cell Line	Oncogenes	
	KRAS	BRAF
AsPC-1	G12D	wt
BxPC-3	wt	V487-P492>A
Capan-1	G12V	wt

wt—wild-type.

## Abbreviations

PDAC	pancreatic ductal adenocarcinoma
PBL	peripheral blood lymphocytes
RAS	retrovirus-associated DNA sequences
MAPK	mitogen-activated protein kinase pathway
PI3K	phosphoinositide-3-kinase
AKT	protein kinase B
RAF	v-raf 1 murine leukemia viral oncogene homolog 1
BRAF	v-Raf murine sarcoma viral oncogene homolog B
VEGFR-2	vascular endothelial growth factor receptor-2
PDGFR-b	platelet-derived growth factor receptor-b
FLT3	Fms-related tyrosine kinase 3
c-KIT	cell growth factor receptor Kit
c-MYC	transcription factor Myc
MTS	3-(4,5-dimethylthiazol-2-yl)-5-(3-carboxymethoxyphenyl)-2-(4-sulfophenyl)-2H-tetrazolium
PI	propidium iodide
RT	room temperature
PBS	phosphate-buffered saline
FBS	fetal bovine serum
